# Association Between Mental Imagery and Change of Direction Performance in Young Elite Soccer Players of Different Maturity Status

**DOI:** 10.3389/fpsyg.2021.665508

**Published:** 2021-06-10

**Authors:** Dorsaf Sariati, Hassane Zouhal, Raouf Hammami, Cain C. T. Clark, Ammar Nebigh, Mokhtar Chtara, Anthony C. Hackney, Nizar Souissi, Urs Granacher, Omar Ben Ounis

**Affiliations:** ^1^Higher Institute of Sport and Physical Education of Ksar-Said, University of La Manouba, Tunis, Tunisia; ^2^Tunisian Research Laboratory, Sport Performance Optimization, National Center of Medicine and Science in Sports (CNMSS), Tunis, Tunisia; ^3^M2S (Laboratoire Mouvement, Sport, Santé)-EA 1274, University of Rennes, Rennes, France; ^4^Research Laboratory: “Education, Motricity, Sports and Health” (UR 15JS01), Higher Institute of Sport and Physical Education of Sfax, University of Sfax, Sfax, Tunisia; ^5^Department of Exercise and Sport Science, The University of North Carolina at Chapel Hill, Chapel Hill, NC, United States; ^6^Centre for Intelligent Healthcare, Coventry University, Coventry, United Kingdom; ^7^Physical Activity, Sport, and Health, UR18JS01, National Observatory of Sport, Tunis, Tunisia; ^8^Division of Training and Movement Sciences, University of Potsdam, Potsdam, Germany

**Keywords:** mental imagery, football, maturation, speed, adolescents

## Abstract

Previous studies have not considered the potential influence of maturity status on the relationship between mental imagery and change of direction (CoD) speed in youth soccer. Accordingly, this cross-sectional study examined the association between mental imagery and CoD performance in young elite soccer players of different maturity status. Forty young male soccer players, aged 10-17 years, were assigned into two groups according to their predicted age at peak height velocity (PHV) (Pre-PHV; *n* = 20 and Post-PHV; *n* = 20). Participants were evaluated on soccer-specific tests of CoD with (CoDBall-15m) and without (CoD-15m) the ball. Participants completed the movement imagery questionnaire (MIQ) with the three- dimensional structure, internal visual imagery (IVI), external visual imagery (EVI), as well as kinesthetic imagery (KI). The Post-PHV players achieved significantly better results than Pre-PHV in EVI (*ES* = 1.58, large; *p* < 0.001), CoD-15m (*ES* = 2.09, very large; *p* < 0.001) and CoDBall-15m (*ES* = 1.60, large; *p* < 0.001). Correlations were significantly different between maturity groups, where, for the pre-PHV group, a negative very large correlation was observed between CoDBall-15m and KI (*r* = –0.73, *p* = 0.001). For the post-PHV group, large negative correlations were observed between CoD-15m and IVI (*r* = –0.55, *p* = 0.011), EVI (*r* = –062, *p* = 0.003), and KI (*r* = –0.52, *p* = 0.020). A large negative correlation of CoDBall-15m with EVI (*r* = –0.55, *p* = 0.012) and very large correlation with KI (*r* = –0.79, *p* = 0.001) were also observed. This study provides evidence of the theoretical and practical use for the CoD tasks stimulus with imagery. We recommend that sport psychology specialists, coaches, and athletes integrated imagery for CoD tasks in pre-pubertal soccer players to further improve CoD related performance.

## Introduction

The concept of children and adolescents participating in youth soccer training has received growing interest among researchers, clinicians and practitioners over recent years. In this context, knowledge of when to apply an appropriate training stimulus during long-term athlete development is essential for effective soccer programming and improving athletic performance (Lloyd et al., 2013; McNarry and Jones, 2014; Hammami et al., 2018). However, major morphological and neural changes occur with growth and maturation (Malina et al., 2004; Hammami et al., 2016) which make it difficult to identify the exact timing to introduce and promote specific physical qualities. Accordingly, differences in timing and tempo of youth physical development have to be considered when introducing specific soccer training stimuli (Sariati et al., 2021). In particular, change of direction (CoD) speed represents a major performance determinant in team sports such as soccer (Sheppard and Young, 2006; Chaouachi et al., 2012; Chaabene et al., 2018; Hammami et al., 2018). The question arises as to the optimal timing of introducing CoD exercises in youth soccer training. In prepubescent soccer players, Lupo et al. (2019) postulated that the promotion of CoD training is more effective than soccer-specific training to improve linear sprint and CoD performances with ball possession.

Moreover, during the completion of CoD activities, athletes process temporal and spatial stimuli of variegated intricacy (for instance, ranging from uncomplicated tasks, such as monitoring ball flight path, to more detailed tasks, requiring interpretation and tracking of numerous team mates and opposition), within various sporting environments (such as structured attacking plays vs. reacting to an unexpected rebound with time or information limitations), to make appropriate decisions (McNeil et al., 2019). Accordingly, it is essential for field sports coaches and psychology specialists to understand the psychological factors that can contribute to CoD performance. One psychological technique proposed to rehearse perceptual-cognitive abilities, game tactics, and reactive tasks CoD is imagery (McNeil et al., 2019; Taneja et al., 2019). However, to the best of our knowledge, no prior study has considered the relationship between imagery and preplanned CoD task.

In a sporting context, imagery was considered as the condition in which a person imagines themselves performing abilities (Watt et al., 2002). Visual (what the athlete sees) and kinesthetic (what the athlete feels during performing a movement) are the two most frequently used mentally modalities of generating images (Hall, 2001).

Several studies have shown that imagery performance is influenced, in a similar manner to physical performance, by neurological activation and psychological demands (task difficulty, temporal regularities, and programming rules) (Jeannerod, 2006; Guillot et al., 2012). In addition, imagery may be difficult to utilize in CoD task performance, because imagery necessitates voluntary effort to envisage a specific scenario (Wyatt and Domercant, 2012). Thus, creating a mental image of an unforeseeable action is somewhat contradictory, given that there is no inherent environmental volatility in imagery. Indeed, the individual must create or manifest the image of their own accord (Spittle and Morris, 2007; McNeil et al., 2019).

Various studies have investigated the effect of mental imagery on CoD speed and agility performance in various physical activities requiring CoD (McNeil et al., 2019; Taneja et al., 2019). In this context, Taneja et al. (2019) showed that the combination of mental imagery training with plyometrics yields better and added effects on vertical jump and agility than the intervention involving plyometrics and mental imagery training alone. However, McNeil et al. (2019) speculated that mental imagery training is not effective for all factors of perceptual-motor performance. Hence, a clear understanding of how mental imagery affect CoD speed performance, will provide practitioners with important information testing and training designs.

In fact, in soccer, to enhance athletes’ physical CoD speed outcomes, several mental performance coaches and trainers use motor imagery-based programs during training process. In soccer, using sport-specific tasks, positive effects have been identified from imagery training for visual search behavior, tactical awareness and anticipation (Robin et al., 2007). Results from other studies have indicated limited or no effects of imagery training for sport-specific perceptual-cognitive skills (Munroe-Chandler et al., 2005; Post et al., 2018). Furthermore, regarding youth elite soccer players, it was suggested that these qualities should be prioritized within the training process during childhood and adolescence (Lloyd and Oliver, 2012; Van Hooren and De Ste Croix, 2020).

Across maturation, increases in body mass, stature, and sprint speed are evidenced (Ford et al., 2011; Lloyd and Oliver, 2012; Lloyd et al., 2013; Rayan et al., 2018). As results, during CoD tasks, differences in performance can occurred (Lloyd et al., 2013; Fiorilli et al., 2016). Therefore, it would be interesting to investigate whether maturation and growth influence relationships between predictors of specific imagery (i.e., external visual imagery (EVI), internal visual imagery (IVI), and kinesthetic imagery (KI) indices) and pre-planned CoD (i.e., CoD speed task).

Additionally, little evidences exist indicating that training programs should be adapted considering periods of maturation. This seems very important since the recent scientific debate on the trainability of youth, the timing of youth CoD speed training and the potential existence of “golden periods of adaptation” (Lloyd et al., 2013; McNarry and Jones, 2014; Hammami et al., 2018). To optimize youth CoD training programs, investigations of different psychological determinants of a pre-planned CoD tasks (i.e., visual and kinesthetic indices) need to be conducted. Additionally, given the differing experimental methods in the current literature, there is a dearth if evidence pertaining to the long-term development of key factors of CoD in youth soccer players and the effect of maturation and the period of peak-height-velocity (PHV) may exert on these psychological determinants.

Thus, the aim of this research was to identify the associations between CoD test with and without the ball and movement imagery ability in youth football players at different maturity statues. Assuming a relatively robust motor performance level in youth athletes across the different neuromuscular capabilities (i.e., CoD speed; Hammami et al., 2018; Sariati et al., 2021), we expect a stable rank order and thus high correlation coefficients between measures of CoD speed and mental imagery (Robin et al., 2007; Lloyd et al., 2013; Fiorilli et al., 2016). Finally, according to the specific pediatric literature (Lloyd et al., 2013; McNarry and Jones, 2014; Hammami et al., 2018), we hypothesized the largest associations to occur in the postpubertal compared with the prepubertal group due to a maturity related increases in proficiency level and thus robustness in motor performance.

## Materials and Methods

### Sample

Forty young soccer players between (12 defenders, 16 mid-fielders and 12 strikers) aged 10 17 years volunteered to participate in this study. They were all team soccer players from the same club in the first division Tunisian soccer league. On average, the players trained ten months per year with five training sessions and one match per week. For all participants, a practical method for predicting years from peak-height-velocity (PHV) as a measure of maturity offset was applied using the following equation (Moore et al., 2015):

Maturityoffset=-7.999994+(0.0036124×age(years)×height(cm))

This equation has previously been validated for boys and presents a standard error of the estimate (SEE) reported as 0.542 years (Moore et al., 2015) and is a non-invasive and practically approved method to predict years from PHV as a measure of maturity offset using anthropometric variables. Therefore, a maturity offset of –1.0 indicates that the player was measured 1 year before this peak velocity, a maturity offset of 0 indicates that the player was measured at the time of this peak velocity, and a maturity offset of + 1.0 indicates that the athlete was measured 1 year after this peak velocity (Mendez-Villanueva et al., 2011; Hammami et al., 2018). Therefore, players were assigned into two groups (pre-PHV, *n* = 20 and post-PHV, *n* = 20).

Participants’ assent and parental written informed consent were obtained in accordance with the latest version of the Declaration of Helsinki. Prior to study participation, all players received a medical examination and were fully informed about the experimental procedures, inherent risks and benefits of the study. The study design was approved by the Ethics Committee of the National Centre of Medicine and Science of Sports (Tunis, Tunisia).

### Sample Size

A minimum sample size of 30 was determined from an *a priori* statistical power analysis using G^∗^Power (Version 3.1, University of Dusseldorf, Germany) (Faul et al., 2009). The power analysis was computed with an assumed power of 0.95, an alpha level of 0.01, and a moderate effect size of 0.51 for our primary outcome, 15-m change of direction run (CoD-15m) (Sariati et al., 2021). The analysis revealed that a total sample size of *N* = 38 would be sufficient to detect medium-sized group-by-time interaction effects. Accordingly, 40 participants were enrolled to account for potential drop-outs due to injuries.

### Procedures

Players were tested at the first half of the competitive season. One week prior to the start of the study, all players familiarized with all study materials, procedures and technique of CoD tests (two sessions/week). During this session, anthropometric measurements were taken. Using a wall-mounted stadiometer, height was measured to the nearest 0.1 cm and body mass to the nearest 0.1 kg on a digital scale (OHAUS, Florhman Park, NJ, United States). The body-mass-index (BMI) was calculated as mass per height squared (kg/m^2^). Using a Harpenden caliper (Baty International, West Sussex, United Kingdom), two skinfold thicknesses (triceps and sub-scapular) were measured in triplicate to determine body-fat percentage (Slaughter et al., 1988).

Before testing, 10-min standardized warm-up was performed by the players. Two trials of the CoD test (with and without ball) were realized by the players on randomized order on three non-consecutive test days. Intra-class correlation coefficients (ICC) and standard error measurement (SEM) were determined. A two-way mixed, average score, ICC model was used, where *k* fixed rates were defined and each participant was measured by the *k* raters, computationally defined as Shrout and Fleiss convention ICC (3, *k*) (Shrout and Fleiss, 1979; Weir, 2005).

### Change of Direction (CoD) Tests

#### 15-m Change of Direction Run (CoD-15m)

**FIGURE 1:**
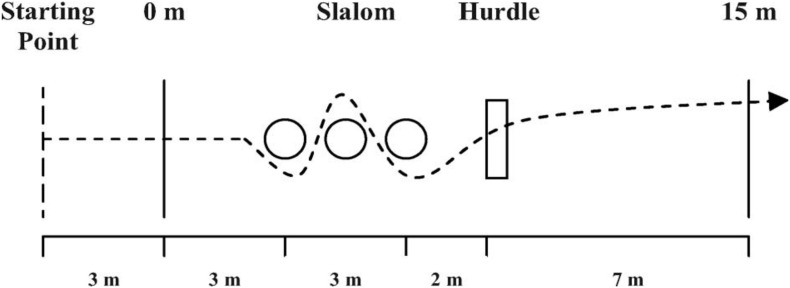
Schematic representation of the CoD test.

In this test, players’ time (Mujika et al., 2009) was measured using two photo-cell gates (Brower Timing Systems, Salt- Lake City, UT, United States, accuracy of 0.01 s). As depicted in Figure 1, players departed running 3 m behind the initial set of gates. Players performed 3 m of straight running, entered a 3-m slalom section marked by three aligned sticks (1.6 m of height) placed 1.5 m apart, and then cleared a 0.5 m hurdle placed 2 m beyond the third stick. Finally, players ran 7 m to break the second set of photocell gates, which stopped the timer. All player completed two maximal CoD-15m interspersed with 3 min of passive recovery, and the fastest time realized was recorded.

#### 15-m Change of Direction Run With Ball (CoDBall-15 m)

This test was similar to the CoD-15m, but players were required to dribble a ball while performing the test (Mujika et al., 2009). After the slalom section of the test the player freely kicked the ball toward either of two small goals placed diagonally 7 m on the left and the right sides of the hurdle, and sprinted to the finish line. Each player performed two maximal CoDBall-15m interspersed with 3 min of passive recovery, and the fastest time achieved was kept for analysis.

#### Movement Imagery Questionnaire 3 French Version (MIQ-3f)

The MIQ-3 is a questionnaire that measures self-assessment (Williams et al., 2012). It contains 12 items assessing the participant’s ability to imagine four movements (arm abduction ad adduction, hip flexion when standing, knee elevation and jumping) after the execution of the movement, through internal visual imagery (IVI), external visual imagery (EVI), and kinesthetic imagery (KI) modalities. After having evaluated the difficulty of the three imaging modalities; EVI, IVI and KI according to the 7-point scales ranging from 1 (very difficult to see/feel) to 7 (very easy to see/feel), an average score ranging from 1 to 7 is calculated for each participant and each modality. The higher score proving a higher motor imagery capacity.

The MIQ-3 was translated into a French version (MIQ-3f) by Robin et al. (2020). Before test session, it was well explained to the participants the descriptions of perspectives of EVI (“try to visually imagine the movement from an external point of view, as if you are watching on TV or from a third person point of view”), IVI (“try to visually imagine the movement from an interior point of view or a first person perspective, it is about visualizing the action through your own eyes”), and KI (“trying to feel the movement actually produced, which will generate a sensation of contractions in the involved muscles or feeling an object with which your body comes into contact ”) (Williams et al., 2012). Players completed the MIQ-3f in a quiet place, under the supervision of the same examiner and in the same standardized conditions. The MIQ-3 demonstrated good internal reliability for each subscale, with ICC values of 0.81 (IVI), 0.89 (EVI), and 0.89 (KI), and AVE values of 0.51 (IVI), 0.66 (EVI), and 0.67 (KI) (Williams et al., 2012). Mean scores and standard deviations of the three- dimensional structure of the MIQ-3f (IVI, EVI, and KI) was previously analyzed by Robin et al. (2020).

The intraclass correlation coefficients (ICC) was *r* = 0.87 for IVI, *r* = 0.86 for EVI, and *r* = 0.88 for KI items (*n* = 172; *p* < 0.05), thus approving a high degree of repeatability above time.

### Statistical Analysis

Data were presented as group mean values and standard deviations (SD). Normal distribution of data was assessed, and confirmed, using the Shapiro-Wilk test. Between-group (i.e., pre-PHV and post-PHV) differences in measures of anthropometry, pre-planned CoD tests (CoD-15m and CoDBall-15m) and the three-dimensional structure of the MIQ-3f (IVI, EVI, and KI) were computed using a 2-sided *t*-test for independent samples.

Additionally, Cohen’s d effect sizes (ES) for differences in studied variables between corresponding groups were calculated, and they were interpreted using the following qualitative descriptors: trivial (< 0.2), small (0.2–0.6), moderate (0.6–1.2), large (1.2–2.0), and very large (2.0–4.0) (Hopkins et al., 2009), as is common place in sports performance literature.

Intraclass correlation coefficients (ICC) and coefficients of variation (CV) were computed to assess relative and absolute test-retest reliability (Hopkins, 2005). The strength of associations was reported with correlation coefficients (*r*-value), level of significance (*p*-value), and the amount of variance explained (*r*^2^-value). The magnitude of the correlations was determined using the modified scale proposed by Hopkins (2019); *r* < 0.1, trivial; 0.1–0.3, small; > 0.3–0.5, moderate; > 0.5–0.7, large; > 0.7–0.9, very large; > 0.9, nearly perfect; and 1 perfect., Average z-transformed correlation coefficients (*rz*-values) were calculated using the Fisher r-to-z transformation. Accordingly, these *rz*-values were used to compute differences in correlation coefficients relative to players’ maturity status (Kenny, 1987). The corresponding formula is: *z* = [*z*1. *z*2)/(1/(*n*1. 3) + 1/(*n*2. 3)].”

Stepwise linear regression analyses were calculated to identify the multivariate relationships between independent variables (predictors; IVI, EVI, and KI) and dependent variables (CoD-15m and CoDBall-15m). For the purpose of the interpretation of explained variance, the coefficient of multiple correlation (R), and the coefficient of determination (percentage of explained variance) were calculated. In order to identify the partial contribution of each predictor to overall multiple regression calculation, the standardized- and non-standardized-regression-coefficients were reported.

Before multiple regressions were performed, the predictors were checked for multicollinearity by calculating the variance inflation factor (VIF), and those variables with a VIF larger than 5 were not included as predictors in the specific multiple regression calculation (Thompson et al., 2017). The significance level was set, *a priori*, at alpha (α) < 0.05. All analyses were performed using Statistica^®^ v.13.0 (Dell Inc., Palo Alto, CA, United States).

## Results

### Reliability Analyses

The test–retest reliability analyses for the CoD (CoD-15m and CoDBall-15m) tests were calculated. Reliability measures (ICC) ranged from 0.93 to 0.97, while percentage values for coefficients of variation ranged from 2.43 to 2.51% and standard error of measurement (SEM) ranged from 0.02 to 0.04. Analyses revealed no significant differences between the performances noted during the two trials for the pre-planned CoD measured variables.

### Between-Group Differences in Anthropometric Characteristics

The post-PHV group was more advanced in experience, age, maturity offset, height, and body mass than pre-PHV (*p* < 0.001; *ES* = 6.24, *ES* = 17.53, *ES* = 12.33, *ES* = 2.14, and *ES* = 1.90; respectively, large to very large). Inversely, the pre-PHV group was better than post-PHV in BMI (*p* < 0.005, *ES* = 0.74, moderate) and body fat (*p* < 0.001, *ES* = 1.00, moderate; Table 1).

**TABLE 1:** Participants’ anthropometric characteristics by player’s maturity status.

	**Pre-PHV (*n* = 20)**	**Post- PHV (*n* = 20)**	**Total (*n* = 40)**
Experience (years)	2.4 ± 0.5	7.4 ± 1.1**	4.9 ± 2.7
Age (years)	10.9 ± 0.4	17.5 ± 0.3**	14.2 ± 3.3
Maturity offset (years)	−1.4 ± 0.3	3.2 ± 0.5**	0.8 ± 2.4
Height (cm)	164.9 ± 4.8	176.8 ± 6.5**	170.9 ± 8.2
Body mass (Kg)	54.6 ± 5.9	67.1 ± 7.5**	60.9 ± 9.2
BMI (Kg.m^–2^)	20.1 ± 1.9*	21.4 ± 1.9	20.7 ± 2.0
Body fat (%)	10.1 ± 2.0**	12.4 ± 2.6	11.2 ± 2.6

Significant difference: *: p < 0.005; **: p < *0.001. Values are means and standard deviations (SD), BMI*, *body mass index; PHV*, *peak height velocity.*

### Between-Group Differences in Measures of Movement Imagery Ability and CoD

The post-PHV players achieved significantly better results than pre-PHV in EVI (*ES* = 1.58, large; *p* < 0.001), CoD-15m (*ES* = 2.09, very large; *p* < 0.001) and CoDBall-15m (*ES* = 1.60, large; *p* < 0.001; Table 2).

**TABLE 2:** Performance variables for movement imagery questionnaire for each item (IVI, EVI, and KI), and the CoD performances (CoD-15m and CoDBall-15m) according to maturity status.

	**Pre-PHV (*n* = 20)**	**Post-PHV (*n* = 20)**	**Cohens’*d***	**Total (*n* = 40)**
IVI	5.23 ± 0.76	5.56 ± 0.95	0.39	5.39 ± 0.87
IVI-1	5.20 ± 1.24	5.45 ± 1.00	0.23	5.33 ± 1.12
IVI-2	5.30 ± 0.92	5.55 ± 1.32	0.23	5.43 ± 1.13
IVI-3	5.30 ± 0.92	5.75 ± 1.16	0.44	5.53 ± 1.06
IVI-4	5.10 ± 0.90	5.50 ± 1.05	0.42	5.30 ± 1.02
EVI	4.88 ± 0.69	5.86 ± 0.58**	1.58	5.37 ± 0.80
EVI -1	4.95 ± 1.00	5.75 ± 0.79**	0.91	5.35 ± 0.98
EVI -2	5.00 ± 1.08	5.85 ± 0.81**	0.91	5.43 ± 1.03
EVI -3	4.85 ± 0.81	5.80 ± 0.83**	1.19	5.33 ± 0.94
EVI-4	4.70 ± 0.98	6.05 ± 0.76**	1.58	5.38 ± 1.10
KI	4.91 ± 1.17	5.46 ± 1.01	0.52	5.18 ± 1.11
KI-1	4.80 ± 1.47	5.55 ± 1.28	0.56	5.18 ± 1.41
KI-2	4.90 ± 1.17	5.35 ± 1.23	0.38	5.13 ± 1.20
KI-3	5.10 ± 1.25	5.60 ± 1.10	0.44	5.35 ± 1.19
KI-4	4.85 ± 1.42	5.30 ± 1.17	0.35	5.08 ± 1.31
CoD-15m (s)	3.22 ± 0.25	2.80 ± 0.15**	2.09	3.01 ± 0.30
CoDBall-15m (s)	4.09 ± 0.37	3.65 ± 0.15**	1.60	3.87 ± 0.36

**Significant difference: p < *0.001. Values are means and standard deviations (SD); Items notched on a scale of 1–7, with superior scores designating greater imagery ability. *IVI*, *internal visual imagery; EVI*, *external visual imagery; KI*, *kinesthetic imagery; PHV*, *peak height velocity.**

### Correlations Between Measures of CoD (CoD-15m and CoDBall-15 m) With the Three-Dimensional Structure of the MIQ-3f (IVI, EVI, and KI)

Results showed a significant negative moderate strength correlation between CoD-15m and IVI (*r* = –0.41, *p* = 0.009) and KI (*r* = –0.47, *p* = 0.002) and large negative correlation with EVI (*r* = –0.66, *p* = 0.001) in the whole sample. Furthermore, negative moderate correlation was observed between CoDBall-15m and EVI (*r* = –0.50, *p* = 0.001) and negative large correlation with KI (*r* = –0.68, *p* = 0.001). For the pre-PHV group, a very large negative correlation was revealed between the CoDBall-15m and KI (*r* = –0.73, *p* = 0.001). For the post-PHV group, large negative correlations were observed between CoD-15m and IVI (*r* = –0.55, *p* = 0.011), EVI (*r* = –062, *p* = 0.003), and KI (*r* = –0.52, *p* = 0.020). Finally, a large negative correlation of CoDBall-15m with EVI (*r* = –0.55, *p* = 0.012) and very large correlation with KI (*r* = –0.79, *p* = 0.001) were also observed (Table 3).

**TABLE 3:** Correlations between studied variables in forty elite youth soccer players according to their maturity status (pre-PHV and post-PHV).

	**Whole Sample (N = 40)**							
	**IVI**		**EVI**		**KI**			
	**r (r^2^)**	**r_*z*_**	**r (r^2^)**	**r_*z*_**	**r (r^2^)**	**r_*z*_**	**μr (r^2^)**	**μr_*z*_**
CoD-15m	–0.41** (0.17)	0.44	–0.66** (0.43)	0.78	–0.47** (0.22)	0.51	–0.51 (0.27)	0.58
CoDBall-15 m	–0.30 (0.09)	0.31	–0.50** (0.25)	0.55	–0.68** (0.47)	0.83	–0.49 (0.27)	0.56
μr (r^2^)	–0.36 (0.13)	–	–0.58 (0.34)	–	–0.58 (0.35)	–	–0.50 (0.27)	–
μrz	–	0.38	–	0.66	–	0.67	–	0.57

	**Pre-PHV (n = 20)**							
	**IVI**		**EVI**		**KI**			
	**r (r^2^)**	**r_*z*_**	**r (r^2^)**	**r_*z*_**	**r (r^2^)**	**r_*z*_**	**μr (r^2^)**	**μr_*z*_**

CoD-15m	–0.31 (0.10)	0.32	–0.29 (0.08)	0.30	–0.41 (0.17)	0.44	–0.34 (0.12)	0.35
CoDBall-15 m	–0.26 (0.07)	0.27	–0.07 (0.01)	0.07	–0.73** (0.53)	0.93	–0.35 (0.20)	0.42
μr (r^2^)	–0.29 (0.08)	–	–0.18 (0.04)	–	–0.57 (0.35)	–	–0.34 (0.16)	–
μrz	–	0.30	–	0.18	–	0.69	–	0.38

	**Post-PHV (n = 20)**							
	**IVI**		**EVI**		**KI**			
	**r (r^2^)**	**r_*z*_**	**r (r^2^)**	**r_*z*_**	**r (r^2^)**	**r_*z*_**	**μr (r^2^)**	**μr_*z*_**

CoD-15m	–0.55* (0.31)	0.62	–0.62** (0.39)	0.73	–0.52* (0.27)	0.58	–0.56 (0.32)	0.64
CoDBall-15 m	–0.26 (0.02)	0.27	–0.55* (0.30)	0.62	–0.79** (0.63)	1.07	–0.53 (0.32)	0.65
μr (r^2^)	–0.41 (0.17)	–	–0.58 (0.34)	–	–0.66 (0.45)	–	–0.54 (0.32)	–
μrz	–	0.45	–	0.68	–	0.83	–	0.65

***–*,**not applicable; *p < 0.05; **p < *0.01; r*, *r-value; r_*z*_*, *r_*z*_-value;*μ, *Mean; r^2^, coefficient of determination which was calculated by squaring the r-value; IVI, internal visual imagery; EVI, external visual imagery; KI, kinesthetic imagery.*

#### Regression Analyses of CoD (CoD-15m and CoDBall-15 m) and the Three-Dimensional Structure of the MIQ-3f (IVI, EVI, and KI)

Statistical analyses showed that in the whole sample, the single best predictor of the CoD-15m was performance in the EVI with an explained variance of 44% (*p* = 0.001). For the CoDBall-15m, the KI and the EVI performances explained 47% (*p* = 0.001) and 53% (*p* = 0.04) of the variance of performance, respectively. For the pre-PHV group, the best predictor of the CoDBall-15m was the KI performance explained 53% of the variance (*p* = 0.001).

In the post-PHV group, for the CoD-15m, the EVI performance explained 39% (*p* = 0.003) of the variance of performance. The best predictor of the CoDBall-15m was the KI performance with an explained variance of 63% (*p* = 0.001; Table 4).

**TABLE 4:** Stepwise linear regression analyses with measures of the CoD (CoD-15m and CoDBall-15m), as criterion variable and the three- dimensional structure of the MIQ-3f (IVI, EVI, and KI), as predictor variables in forty elite youth soccer players according to their maturity status.

	**Model**		**UC**		**C**	***t***	***p***	***R*^2^ [R^2^]**
			**B**	**SE**	***β***			
**Whole Sample (*n* = 40)**								
CoD-15m	1	(Constant)	4.32	0.24		17.84	0.001	
		**EVI**	–0.24	0.05	–0.66	–5.45	0.001	0.44 [0.42]
CoDBall-15 m	2	(Constant)	5.48	0.29		19.01	0.001	
		**KI**	–0.19	0.04	–0.58	–4.67	0.001	0.47 [0.45]
		**EVI**	–0.12	0.06	–0.27	–2.18	0.04	0.53 [0.50]

**Pre-PHV (*n* = 20)**								
CoD-15m		NA	NA	NA	NA	NA	NA	NA
CoDBall-15 m	1	(Constant)	5.24	0.26		20.02	0.001	
		**KI**	–0.23	0.05	–0.73	–4.50	0.001	0.53 [0.50]

**Post-PHV (*n* = 20)**								
CoD-15m	2	(Constant)	3.74	0.28		13.33	0.001	
		**EVI**	–0.16	0.05	–0.62	–3.37	0.003	0.39 [0.35]
CoDBall-15 m	1	(Constant)	4.27	0.12		37.09	0.001	
		**KI**	–0.11	0.02	–0.79	–5,51	0.001	0.63 [0.61]

*N/A, not applicable; UC, Unstandardized Coefficients; C, Coefficients; SE, Standard Error:*β*, *Beta; p, signification; R^2^, R Square; [R^2^], Adjusted R Square; IVI, internal visual imagery; EVI, external visual imagery; KI, kinesthetic imagery.**

## Discussion

The present study sought to investigate the relationships between the CoD (CoD-15m and CoDBall-15m) tests and cognitive components in youth soccer players of different maturity status. To the best of our knowledge, this is the first study to examine the relationship between the CoD test, with and without the ball, and the three-dimensional structure of the MIQ-3f (IVI, EVI, and KI) in elite youth soccer players.

Overall, moderate to very large correlations were observed between CoD tests and IVI, EVI, and KI. Additionally, there were differences in these associations depending on the players’ maturity status. According to the extant literature (Robin et al., 2007; Lloyd et al., 2013; Fiorilli et al., 2016), it is likely that, with regard to maturation, imagery would be similar to physical stimuli, and improvements in perceptual components would translate into overall CoD performance in the more mature group (i.e., post-PHV group). Indeed, moderate to very large correlations were observed between CoD tests and IVI, EVI, and KI, and these correlation coefficients were significantly different between maturity groups, with larger associations in the post-PHV group compared to the pre-PHV group.

It is well established that the type sport practice may also exert an effect on the use of either internal or external visual perspective, depending on the sport (Hall, 2001; Corrado et al., 2015). Nimmerichter et al. (2016) showed that, performance of reactive CoD improved after 6-weeks of video-based training in young male soccer players, concomitant to a significant association with reaction speed. In addition, a significant association between the total time of the reactive CoD test and the response time to the stimulus was shown in male soccer players (Young and Willey, 2010). In fact, results from various studies have suggested that mental imagery can improve performance in competitive situations and motor tasks, and facilitates learning and motor acquisition (Cumming and Ramsey, 2009; Moreau et al., 2010).

Moreover, previous research has posited that motor control improvement may be maturity related and the magnitude of adaptations could be related to the maturity status (Malina et al., 2004; Granacher et al., 2016; Hammami et al., 2016). In this context, Hammami et al. (2018) demonstrated that moderate to small associations between measures of CoD, speed, and muscle power differ according to the maturity status. These authors speculated that the difference in correlation strength may be attributed to the mental maturation that can be observed as better performances were found in the more mature group. The present results showed that the post-PHV group outperformed the younger groups in the imagery indices and CoD, with and without the ball.

Coordination can show deficits in children in instances where their neuromuscular system is not yet fully matured and their motor skills are still emerging (Payne and Isaacs, 2005; Lloyd et al., 2013; Hammami et al., 2016). Motor control improves with physical maturation (Malina et al., 2004), and, thus, enhanced coordination would likely contribute to improved CoD (Lloyd et al., 2013). Furthermore, Behringer et al. (2011) emphasized that training-induced improvements in muscle strength translated to motor skill performance in children and adolescents. Similarly, CoD (Lloyd et al., 2013; Hammami et al., 2018) has been shown to improve with somatic maturation (Hammami et al., 2018); hence, as children growing through the somatic maturational stages, we expect that the greater pre-planned CoD test performances by older children would be more influenced by imagery indices. Accordingly, the more mature group (post-PHV) may have generated a better image of the stimulus than the pre PHV group, which may make the task more familiar (i.e., direction of the stimulus, how the stimulus appeared on the screen) and/or required movements (i.e., left or right direction). Moreover, because imagery performance is related to CoD performance, the latter may be explained by the fact that it is easier to generate a known task than generating an unknown or unpredictable event during imagery (Borst and Kosslyn, 2008; McNeil et al., 2019; Taneja et al., 2019).

Conversely, the pre-PHV group demonstrated moderate associations between CoD and imagery performances. The rationale for this finding might be attributed to the rapid development of the PHV phase, to efficiently negotiate the CoD demands of soccer, but also the cognitive capability to perceive and react within a very short time frame during play (Hammami et al., 2017, 2018) in this group. That is, the vestibular systems of the pre- and post-PHV groups would have a more developed morphology (i.e., height, mass, limb, and trunk lengths) over time with which to generate important perceptual-motor components (i.e., temporal and spatial unpredictability of the stimulus) in order to optimize their strategies to enhance CoD performance.

The lack of correlation in the less mature group, compared to the more mature group, suggested that not all components of CoD performance (i.e., with and without the ball) were strongly influenced by imagery, supporting that the type of task moderates the effectiveness of imagery (Driskell et al., 1994). Researchers have suggested that perceptual-cognitive skills are improved by the use of imagery (Guillot and Collet, 2005; Jordet, 2005; Smeeton et al., 2013); this, however, does not appear to translate to a CoD task using specific stimuli, where sport-specific perceptual-cognitive skills (i.e., soccer skills) are not employed. Indeed, these differences may support the issues associated with generating a predictable event based upon the conceptual nature of imagery (Paivio, 1985; Munroe et al., 2000; Spittle and Morris, 2007).

The pre-PHV group was not able to improve perceptual skills and performance that could be pre-determined, and, as such, were likely unable to accurately imagine as it pertains to real-game situations. Thus, future research should focus on the longitudinal effect of mental imagery on CoD performance in pre-pubertal soccer players with less developed neuromuscular systems.

Previous studies have not considered the potential influence of maturity status on the relationship between mental imagery and change of direction (CoD) speed in youth soccer. Accordingly, to the authors’ knowledge, the present study represents the first to have examined the association between mental imagery and CoD performance in young elite soccer players of different maturity status. However, notwithstanding the novel addition to the literature, this study is not without limitations. First, the sample size is small in each group (pre- and post-PHV) and, as such, this limits the statistical power and the generalizability of the findings. However, given this is the first work of its’ kind, we hope that it may be used as platform to further develop our understanding of a complex relationship. Secondly, we used a single test of CoD (with and without ball), however, it is noteworthy to mention that a multitude of others exists. Accordingly, while our results provide interesting information for conditioning specialists and coaches, they have to be interpreted with caution and should be verified in further investigations. Finally, in the present study, additional covariates, mediators, and moderators of the relationship between CoD performance and movement imagery ability was not discerned, principally because it out of the scope of the aims of this study.

However, we advocate that further research, considering covariates and exploring mediating and moderating factors, may now be conducted to build on the platform provided by this study.

In addition, soccer-specific training is easily associable to mental imagery and players’ sprint abilities demonstrated during a match. However, no clear evidence has been provided to verify the differential effects of mental imagery on CoD speed performance during different soccer specific training regimens (agility drills vs. training focused on running techniques for sprints) in prepubescent soccer players at different training status and competition levels. Hence, future research in this context is warranted.

## Conclusion

This study provides the first empirical evidence of the theoretical and applied use for the CoD tasks stimulus with imagery. Imagery provided an opportunity to influence CoD performance in the more mature group of youth soccer players. In addition, performance improvements in this group suggest that these young athletes are able to generate an image that realistically captures important CoD performance components necessary for performance improvements.

Based on prior training studies (McNeil et al., 2019; Taneja et al., 2019), and the moderate-to-strong correlations found in the current study, a greater emphasis on prior mental imagery activities and training should be placed on younger soccer players (Pre-PHV) with a less developed neuromuscular system to improve efficacy of CoD performance. In addition, future studies should examine the long-term effects of mental imagery activities included in CoD or soccer-specific training on sport-specific performance outcome measures in soccer players of different maturity and/or expertise level.”

## Data Availability Statement

The raw data supporting the conclusions of this article will be made available by the authors, without undue reservation.

## Ethics Statement

The study was conducted according to the latest version of the Declaration of Helsinki and the protocol was fully approved by the Local Ethics Committee of the National Centre of Medicine and Science of Sports of Tunis (CNMSS) before the commencement of the assessments. Written informed consent to participate in this study was provided by the participants’ legal guardian.

## Author Contributions

DS, OBO, RH, NS, and HZ participated to the conception and design of the study. DS and AN were responsible for testing. DS, MC, CC, UG, and OBO were responsible for data collection and statistical analysis. DS, AH, UG, HZ, and OBO were responsible for writing and finalization of the manuscript. All authors contributed to manuscript and approved the submitted version.

## Conflict of Interest

The authors declare that the research was conducted in the absence of any commercial or financial relationships that could be construed as a potential conflict of interest.
